# Implementation of soft microfingers for a hMSC aggregate manipulation system

**DOI:** 10.1038/micronano.2015.48

**Published:** 2016-02-15

**Authors:** Satoshi Konishi, Shuhei Shimomura, Shuhei Tajima, Yasuhiko Tabata

**Affiliations:** 1 Department of Mechanical Engineering, Ritsumeikan University, 1-1-1 Noji-higash, Kusatsu, Shiga 525-8577, Japan; 2 Graduate School of Science and Engineering, Ritsumeikan University, 1-1-1 Noji-higash, Kusatsu, Shiga 525-8577, Japan; 3 Institute for Frontier Medical Sciences, Kyoto University, Yoshida-shimoadachi, Sakyo-ku, Kyoto 606-8501, Japan

**Keywords:** cellular aggregate, manipulation, pinching, releasing, positioning, pneumatic balloon actuator, soft MEMS, surface modification

## Abstract

This paper describes a pneumatic balloon actuator (PBA) composed of polydimethylsiloxane (PDMS) for cellular aggregate manipulation. We evaluated the ability of the microdevice to manipulate a tiny and sensitive cellular aggregate without causing serious damage. We used human mesenchymal stem cells (hMSCs) for the cellular aggregate. We describe the design, fabrication, characterization and operation of the soft microfingers to pinch and release a spherical hMSC aggregate (*φ*200 μm), and we employed a PBA to serve as an artificial muscle to drive the microfingers. A design of the microfingers in terms of dimensions, generated force and contact conditions was accomplished. The designed dimensions of a single finger were 560 μm×900 μm. In summary, we demonstrate the utility of the surface modification of a fingertip for pinching and releasing a cellular aggregate and describe a manipulation system that was constructed to drive and control the microfingers. The implemented manipulation system, which is composed of microfingers and a positioning mechanism, was tested and verified in a series of operations.

## Introduction

A human interface requires soft and flexible features to accomplish its function while following the deformable shapes of a living body. Various soft and flexible devices have been reported for wearable products^1,2^. It has also become increasingly important to incorporate softness and flexibility into microelectromechanical systems (MEMS), especially those used for biomedical applications such as a neurointerface and in the design of minimally invasive medical instruments^3^.

MEMS were first established on microelectronics technologies using Si as the principal semiconductor material. In addition to hard Si substrates that are commonly used for microelectronics and MEMS, a flexible substrate such as a polyimide circuit board has been widely used in the design of electronics products. A polyimide circuit board is useful for connecting different rigid substrates due to its flexibility. Multielectrodes are used to employ a flexible polymer circuit board as a substrate for a neurointerface.

In addition to softness and flexibility, polymers have additional attractive features as a substrate of MEMS for biomedical applications. A micro channel for micro total analysis systems (μTAS) requires fine structures with optically transparent or electrically insulating properties. A transparent polymer allows for observations of phenomena in a micro channel. Multiple electrodes can be arranged and isolated onto an insulating polymer structure. Various methods have been developed for the manufacturing of polymer microstructures. For example, the molding technique is one of the most common methods used for polymer micromachining. Printing technology such as screen-printing technology has been used for fine patterning on a flexible substrate. In addition, inkjet-printing technology for MEMS has been reported^4,5^, and the roll-to-roll process has attracted attention as a highly productive method^6^.

As one of the most advanced biomedical technologies, tissue engineering shows great potential in both regenerative medicine and drug development^7–12^. In the future, it may be possible to provide personalized medicine using cultured cells based on this technology. However, cultured cells in a dish or chip are prepared in a planar environment, whereas real tissues in the human body consist of three-dimensional cellular structures. Therefore, three-dimensional cellular structures should be used to examine phenomena that can better mimic the actual processes occurring in the human body. Scaffold techniques allow for the laboratory production of three-dimensional cellular structures^9^, and three-dimensional cell cultures such as cellular aggregates have been studied for applications in drug screening^10–12^. In laboratories, cultured cells and tissues are usually handled with conventional pipetting techniques. However, to ensure quality assurance and precise control of a biological product, a specific device is required for the manipulation of tiny and sensitive cellular tissues without inducing damage.

**Figure 1:**
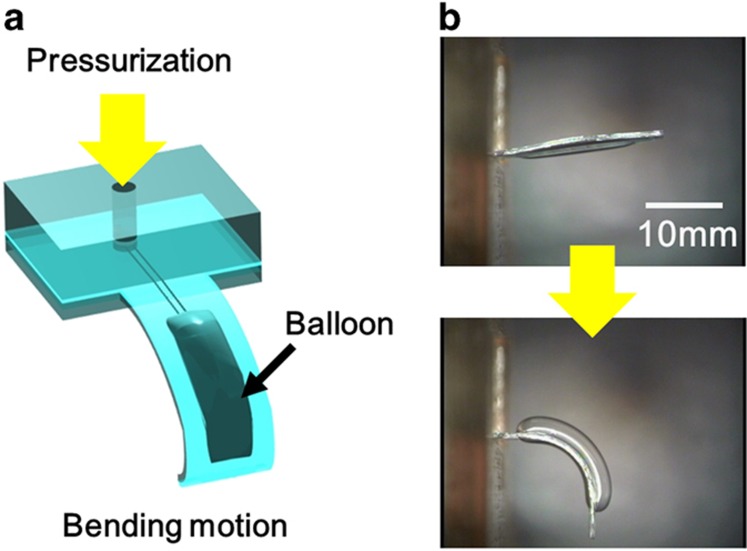
Basic principle of an all PDMS bending PBA consisting of two PDMS films with different thicknesses or material properties: (**a**) schematics; (**b**) photographs.

To this end, devices for culturing and transporting a cellular aggregate have been reported in the field of μTAS^13^. We recently reported a cellular aggregate capture device using fluidic manipulation^14,15^. Other studies have also focused on methods for cell manipulation^16,17^. Compared to the non-contact-type manipulation of cultured cells, soft structure and flexible motion are suitable for the contact manipulation of tiny and fragile living organisms. One of the present co-authors has studied the use of a pneumatic balloon actuator (PBA), which has small, soft and safe features. A PBA uses polydimethylsiloxane (PDMS) as its structural material and uses pneumatic pressure as its safe driving principle^18^. On the basis of this design, the use of soft microfingers driven by the PBA to pinch and release a cellular aggregate was proposed^19^. The proposed microfingers are driven by the PBA, which was developed as an artificial muscle^20^. The polymers provide soft and flexible structures and also showed good compatibility with fluidic MEMS. Among the candidates for the driving principle of actuators, pneumatic actuation shows particular advantages due to its high-force density^21^. Moreover, the combination of an elastic polymer structure and pneumatic actuation enables safe operation. The PBA was first designed to transform the swelling motion of a balloon into a bending motion^20^ and was developed for generations^18,22^. Figure 1 shows the basic principle of the all-PDMS PBA developed as the third generation^18^. Specifically, the third-generation PBA consists of two PDMS films with different thicknesses or material properties. The typical size of a PBA is sub-millimeters in-plane and several tens of micrometers in thickness. A PBA typically generates hundreds of milliNewtons at hundreds of kilopascals.

One of the present co-authors has been continuously working on small, soft and safe MEMS to overcome the current challenges in biomedical applications including neuro-engineering^7,23^, minimally invasive surgery^24^, drug delivery^25^ and tissue engineering^26,27^. For example, transplantation surgery of cellular sheets based on tissue engineering shows good potential to effectively cure inextirpable disease or congenital failure. In fact, for transplantation surgery in a sensitive eye, which is one of the most challenging medical operations because of the narrow surgical space, a novel surgical instrument for the retinal pigment epithelium sheet transplantation was demonstrated using PBA^25,26^. In this paper, we present a novel application of a PBA showing its potential in the abovementioned achievements in biomedical fields.

## Materials and methods

### Preparation of cellular aggregate

We used human mesenchymal stem cells (hMSCs) for the cellular aggregate^28^. Bone-marrow-derived immortalized hMSCs were kindly supplied by Dr Toguchida’s laboratory (Kyoto, Japan)^28^. The hMSCs were cultured in Dulbecco’s modified Eagle’s medium (Invitrogen, Carlsbad, CA, USA) supplemented with 10 vol% fetal calf serum (Thermo, Waltham, MA, USA), penicillin (50 U mL^−1^), and streptomycin (50 U mL^−1^) (standard medium) and then cultured at 37 ℃ in a 95% air/5% carbon dioxide atmosphere. The culture medium was changed every 2 days and the confluent cells were subcultured though trypsinization. Preparation of hMSC aggregates will be described. A PVA sample (degree of polymerization, 1800; saponification, 88 mole%), kindly supplied from Unichika (Tokyo, Japan), was dissolved in phosphate-buffered saline (PBS; 1 wt%). PVA solution was added to each well of round-bottomed (U-bottomed) 96-well culture plate (100 mL per well) and incubated at 37 ℃ for 15 min. Next, the solution was removed by aspiration and the wells were washed twice with PBS (100 mL per well). hMSCs were separately suspended in the standard medium, and the hMSC suspension (2×10^4^ cells mL^−1^, 50 mL per well) was added to the coated wells. After 1 day, hMSC aggregates were formed and used for the next experiment.

### Design and fabrication of microfingers using a PBA

**Figure 2:**
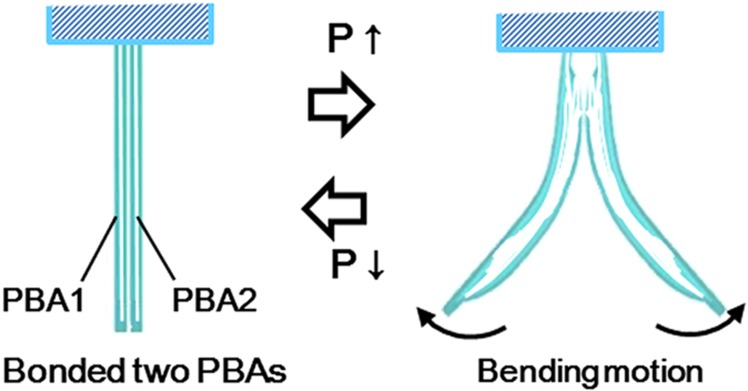
A schematic view of the microfingers composed of two opposing PBAs. Normally, closed microfingers that are open due to the bending motion of the PBAs.

**Figure 3:**
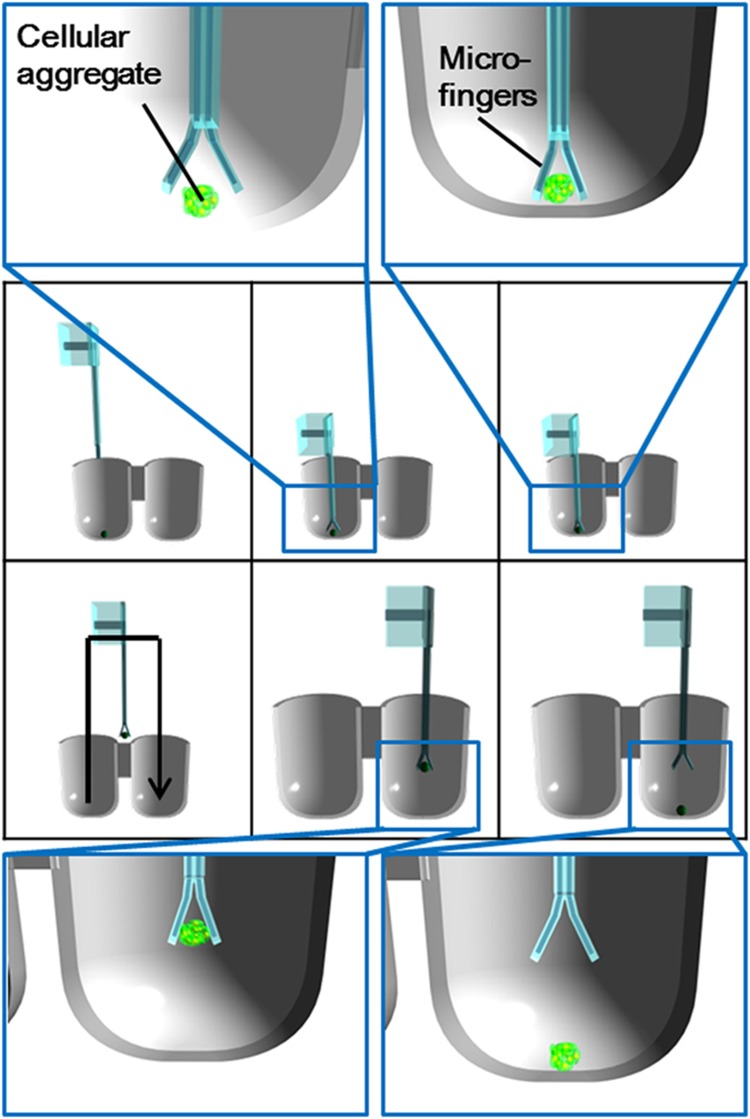
Series of operation for cellular aggregate manipulation. (**a**) Initial state; (**b**) inserting opened microfingers into a well; (**c**) pinching of an object; (**d**) moving to a desired; (**e**) positioning into the desired well; (**f**) releasing of the object.

Microfingers were designed to manipulate tiny and sensitive cellular aggregate without damage to ensure reliable biological products. Figure 2 shows a schematic view of the microfingers. The bending motions of two opposing PBAs facilitate the opening/closing motion of the fingertips. The fingertips close normally and open by pressurization. Figure 3 shows a series of operations: (1) initial state; (2) introducing the microfingers in to a well and opening the fingertips by actuators; (3) pinching of an object by stopping the actuation; (4) moving to a desired well; (5) positioning in a well; and (6) releasing an object by opening the fingertips.

Microfingers were fabricated by considering biocompatibility with the use of microfabrication-friendly materials. The microfingers were designed by taking into account of the size of a cellular aggregate, which was estimated to be 200 μm for this study. Furthermore, the size of the well plate (650185, Greiner-bio-one) was also considered. As a result, the opening gap between opposing microfingers was designed to be 400 μm. A microfinger driven by PBA bends at 30° and 150 kPa based on our typical design and condition. Thick PDMS (8:1 in mixture ratio) and thin PDMS (12:1 in mixture ratio) were spin-coated at 1000 rpm and 3000 rpm, respectively, and were then bonded together to form the PBA. The length and width of each fingertip was designed to be 900 and 560 μm, respectively. A whole microfinger was designed to be 12 mm in length and 1.6 mm in width by taking into account the size, which was 10 mm in depth and 7 mm in diameter.

In our design, the microfingers could be normally closed and opened by the PBA. The pinching force was determined based on the stiffness of the PDMS-based structure, which was later estimated to be several tens of microNewtons.

**Figure 4:**
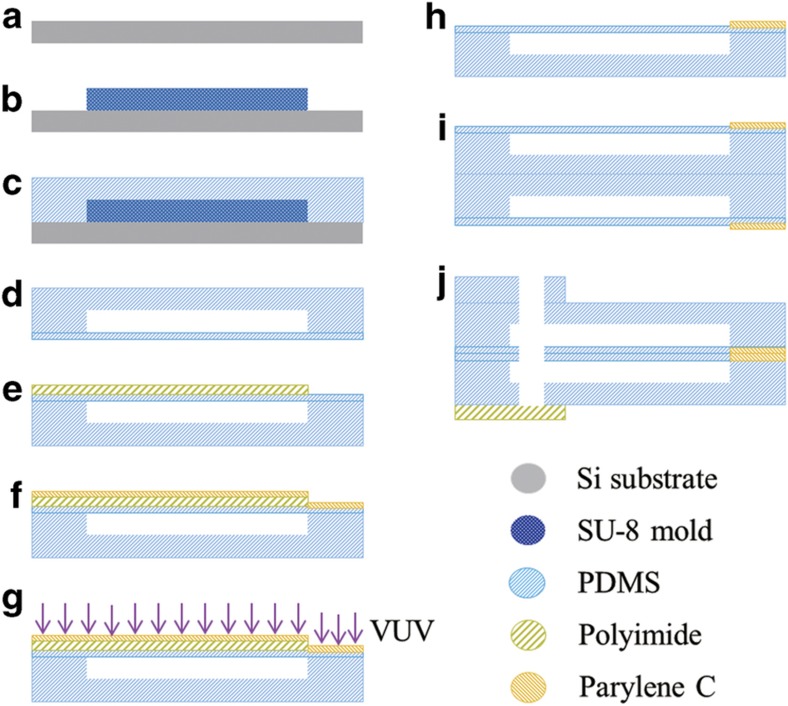
Fabrication process of microfingers. (**a**) Si substrate, (**b**)formation of SU-8 mold, (**c**) spin-coating and thermal-curing of PDMS on SU-8 mold, (**d**) peeling off from SU-8 mold and bonding to thin PDMS film, (**e**) placement of polyimide mask onto the thin PDMS film, (**f**) deposition of parylene C onto fingertips, (**g**) surface hydrophilic treatment by VUV, (**h**) removal of the polyimide mask, to prepare a single microfinger, (**i**) bonding of two microfingers, and (**j**) punching for interconnection.

**Figure 5:**
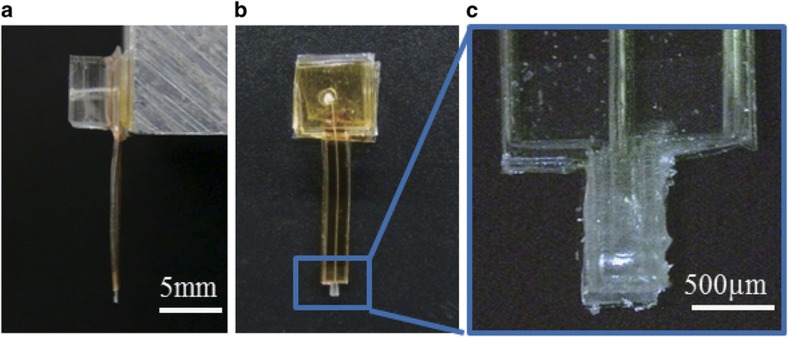
Fabrication results of microfingers. (**a**) Front view, (**b**) side view, (**c**) magnified image of the fingertips. A single finger was 560 μm×900 μm. PBA at the fingertip was 160 μm×600 μm.

Figure 4 shows the fabrication process of the microfingers. First, an SU-8 mold structure was formed on a Si substrate (Figures 4a and b). Next, the PDMS was spin-coated on an SU-8 mold and then thermally cured (Figure 4c). As shown in Figure 4d, the PDMS film was peeled off and pre-cured, and then placed onto another thin PDMS film. The two PDMS films were completely thermally cured so as to be bonded together (Figure 4d). After masking with the polyimide film (Figure 4e), parylene C was deposited on the surface of the fingertip to prevent two opposing fingertips from sticking together (Figure 4f). Sequentially, the surface was exposed to vacuum ultraviolet (VUV) treatment to control its hydrophilicity (Figure 4g). Details of the surface modification of the microfingers are provided in the following sections. Unnecessary parylene C was removed by lift-off together with the polyimide mask (Figure 4h). A single microfinger was prepared at this step. Two microfingers were positioned back-to-back and bonded together (Figure 4i). An interconnection hole was punched to complete the fabrication process (Figure 4j). Figure 5 shows the resulting fabrication of the microfingers; the dimension of a single finger was 560 μm×900 μm.

### Surface treatment of the microfingers

Water-soluble PVA is typically used to hydrophilize a well plate to serve as a culture vessel for a cellular aggregate as aforementioned. A PVA, which is a synthetic resin with strong hydrophilicity, is coated and dried onto the surface. This study used PVA to hidrophilize the surface of the microfingers. Perfluorodecyltrichlorosilane (FDTS) was also used to prepare hydrophobic surface on the PDMS. FDTS was formed on the PDMS surface as follows. For the fluorination, the surface of the PDMS device was modified using the silane-coupling technique with 1H,1H,2H,2H-perfluorododecyltrichlorosilane (FDTS, Sigma-Aldrich, St. Louis, MO, USA). Prior to treatment with the silane-coupling agent, the surface of the PDMS device was first treated with low-pressure oxygen gas plasma or VUV. The OH groups and COOH groups were then generated on the PDMS polymer chains. The activated PDMS device was treated with FDTS at room temperature over 12 h in a vacuum desiccator.

Alternatively, VUV can be used to hydrophilize and clean an object via ultraviolet radiation. The active oxygen formed by the ultraviolet radiation cuts the molecular chains of the irradiated surface layer and generates new functional groups (OH, CHO and COOH) by reacting with the cut molecules. We performed VUV treatment on the parylene C surface. The high hydrophilicity of these functional groups improved the hydrophilicity of the irradiated object.

### System composition

The implemented system is composed of the fabricated microfingers, a probe, an XYZ stage, and a pneumatic system. The probe and XYZ stage were accessories of the manual prober (OYM-90, Oyama, Co., Ltd., Kobe, Hyogo, Japan). The strokes and feeding distances in all directions were 10 mm and 500 μm/rotation, respectively. The motorized positioning stage (XA05A-L2, Kohzu Precision Co., Ltd., Kawasaki, Kanagawa, Japan) was introduced in addition to the manual prober. The stroke and resolution of the positioning system were ±25 mm and 1 μm, respectively. The pneumatic system had a pressure source (AS4P-6, KOBELCO), a regulator (ITV0050, SMC), and an electromagnetic valve (VX240KF, SMC). The present system was manually positioned using a manual positioner; however, use of an automatic position control system is currently being investigated. The behavior of microfingers and the system were observed by two microscopes (PS-1000, SHICOH and VHF-500 F/VH-Z50L, KEYENCE). One was used for observations from the bottom of the dish and the other was for viewing from the side. In our evaluation, a load cell (LVS-5GA, Kyowa Electronic Instruments Co., Ltd., Chofu, Tokyo, Japan) was used to measure the force generated by the microfingers.

### Restoring force measurement of the microfingers

A cultured spherical cellular aggregate reaches up to several hundred micrometers in diameter. The fingertips are opened by 100 μm when the microfingers pinch a *φ*200-μm cellular aggregate. We measured the restoring force generated by bent fingertips made of PDMS. The fingertip was fixed at one edge and was pushed by a load cell (LVS-5GA, Kyowa Electronic Instruments Co., Ltd.) positioned by XYZ stage. The load cell can measure the restoring force generated by the fingertip. As a result, the restoring force was measured to be 30 μN when the fingertip was bent by 100 μm. The restoring force was increased proportional to the deformation of fingertip. The restoring force increases ~45 μN when the microfingers pinch a *φ*300-μm cellular aggregate.

### Evaluation of cell damage

We used lactate dehydrogenase (LDH) activity to quantify the cell damage^29^. The percentage of cells damaged was determined by measuring the assay-determined LDH released relative to a completely lysed sample (sonicated) and a supernatant sample before being subjected to the flow contraction. The LDH assay was performed by LDH Cytotoxicity Detection Kit (Takara Bio Inc., Kusatsu, Shiga, Japan) according to the manufacturer’s instructions.

## Results and discussion

### Basic performance of the microfingers

**Figure 6:**
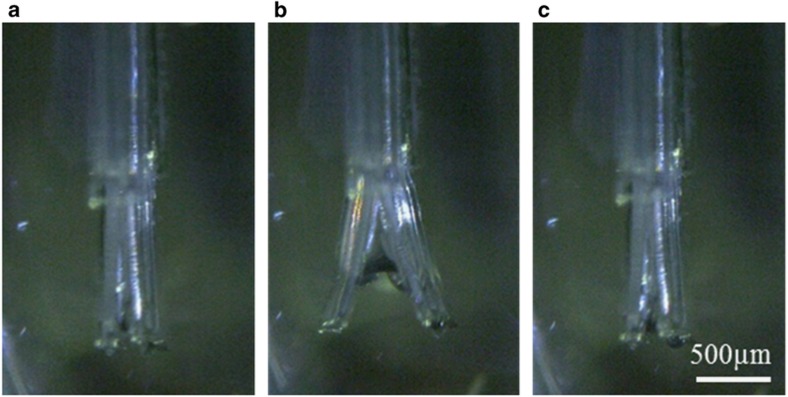
Driving results of microfingers in PBS solution. (**a**) Initial state of microfingers, (**b**) opened fingers by pressurization, and (**c**) closed fingers by decompression.

**Figure 7:**
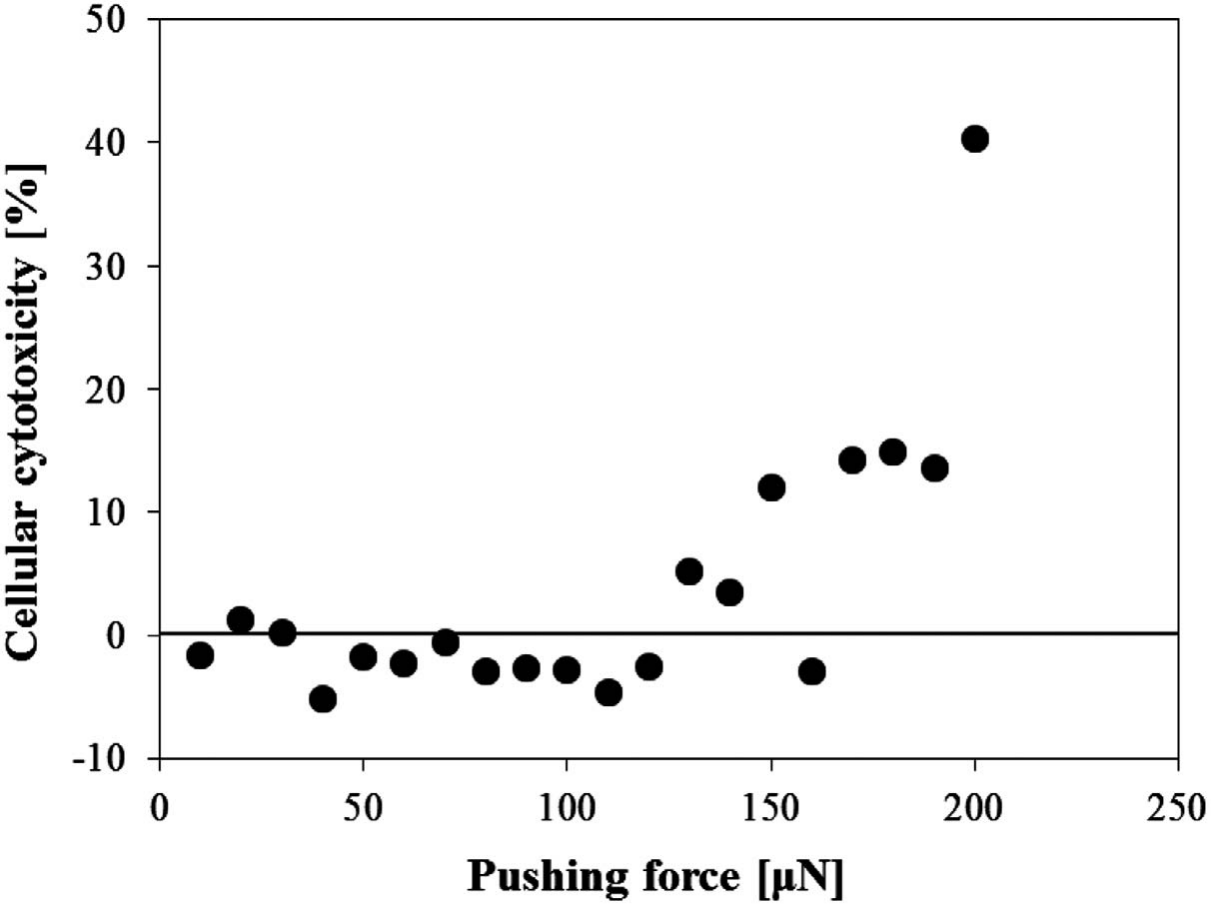
Evaluation result of cellular cytopathy using a LDH assay. The cellular cytotoxicity began to increase at ~130 μN.

First, the pinching of a cellular aggregate was estimated to evaluate the basic performance of the microfingers. Figure 6 shows the operation result of the microfingers. The developed microfingers were driven in the air as well as in PBS. PBA was operated to open the fingertips that were set to be normally closed. A cellular aggregate (that is, the target object) was pinched by the restoring force of the PDMS structure. For characterization, we measured the restoring force of the developed microfingers using a load cell. The pinching force against a *φ*200-μm cellular aggregate was estimated to be 30 μN. Furthermore, we evaluated the damage caused to the cellular aggregate when it was pinched by the microfingers. We applied loads in the range of 10–200 μN to the cellular aggregate and then measured the LDH activity in terms of the cellular cytotoxicity (Figure 7). The LDH assay showed that the cellular cytotoxicity began to increase at a load of 130 μN and tended to increase for larger loads. Consequently, the cytotoxicity can be considered to be sufficiently small because the microfingers are estimated to apply a load of only 30 μN when they pinch a *φ*200-μm cellular aggregate.

### Surface modification for pinching and releasing by the microfingers

We evaluated sequential manipulations involving both pinching and releasing of a cellular aggregate by the microfingers. First, we used the microfingers made of PDMS coated with parylene C without any additional surface modification. When the microfingers were opened for releasing of the cellular aggregate after pinching it by the microfingers, it tended to stick on the microfingers and was difficult to release. We considered that the adhesive strength of the cellular aggregate depended on the hydrophilicity of the contact surface. On one hand, low hydrophilicity would prevent the microfingers from releasing an object. On the other hand, high hydrophilicity of the surface of microfingers would cause difficulty in holding onto the object.

Parylene C was deposited on the surface of the fingertip to prevent the two opposing fingertips from sticking together. We decided to apply a further surface modification to improve the surface condition for both pinching and releasing. Thus, PVA coating was first applied to hydrophilize the surface. In addition, we estimated the adhesive dependence of cellular aggregates on a hydrophobic surface using FDTS as a control.

Table 1 summarizes surface condition dependence of the contact surface on pinching and releasing. First, we focused on PVA, which is coated on the surface to increase the hydrophilicity. The microfingers were introduced into the PVA solution in the open state and then air dried for 15 min. As a result of this treatment process, the contact angles of parylene C surface decreased from 95° to 12°. This manipulation made it more difficult to hold on to the cellular aggregate so that it was easier to release.

As a control, the effects of FDTS coating, which has a higher hydrophobicity, were also estimated. The contact angle of the FDTS-coated surface was 117°. As expected, the FDTS surface showed a similar result to the parylene C surface, in that it was not possible to release the cellular aggregate after holding. Therefore, the PVA treatment was applied to the FDTS surface in the same manner as conducted for the parylene C surface. This process resulted in a decrease of the contact angle of the FDTS surface to 17°, and also made it more difficult for the microfingers to hold on to the cellular aggregate.

These results showed that a surface with high hydrophilicity prevents the microfingers from holding onto the cellular aggregate.

We next focused on parylene C and established intermediate condition between the above treatments. We estimated VUV exposure for the hydrophilic treatment of the parylene C surface. The irradiation time of VUV was set to 150 s. The VUV treatment of the parylene C-coated PDMS resulted in a contact angle of 40°, which enabled the microfingers to effectively pinch and release a cellular aggregate.

### Cellular aggregate manipulation by microfingers

**Figure 8:**
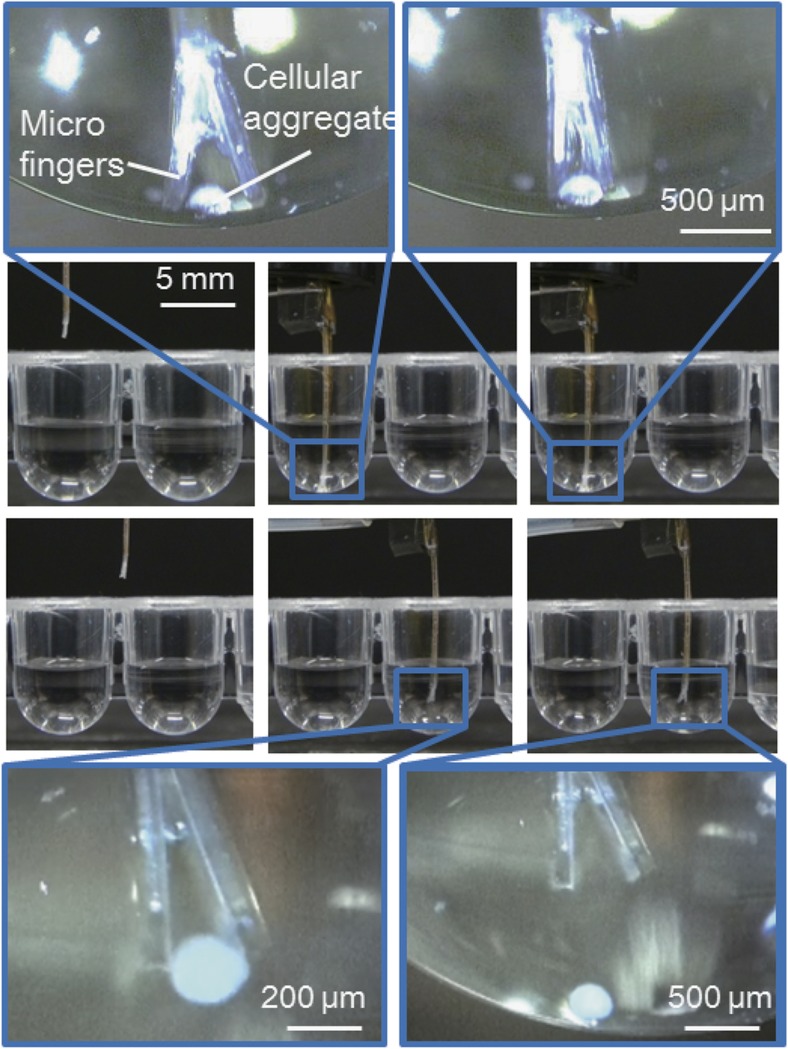
A series of operations of pinching a cellular aggregate (200 μm). Photographs correspond to the illustrations of Figure 2. The cellular aggregate could be pinched, moved, and then released from one well to another target well.

Figure 8 shows a series of operations using the improved microfingers. The microfingers were observed both from the side and the bottom using two microscopes and positioned on an XYZ stage. The magnified photographs in the upper left/right-hand corners of Figure 8 show a *φ* 200-μm cellular aggregate together with the opening/closing the microfingers. The gap between the open fingertips was ~450 μm. Therefore, we could successfully execute the pinching and releasing operation.

**Figure 9:**
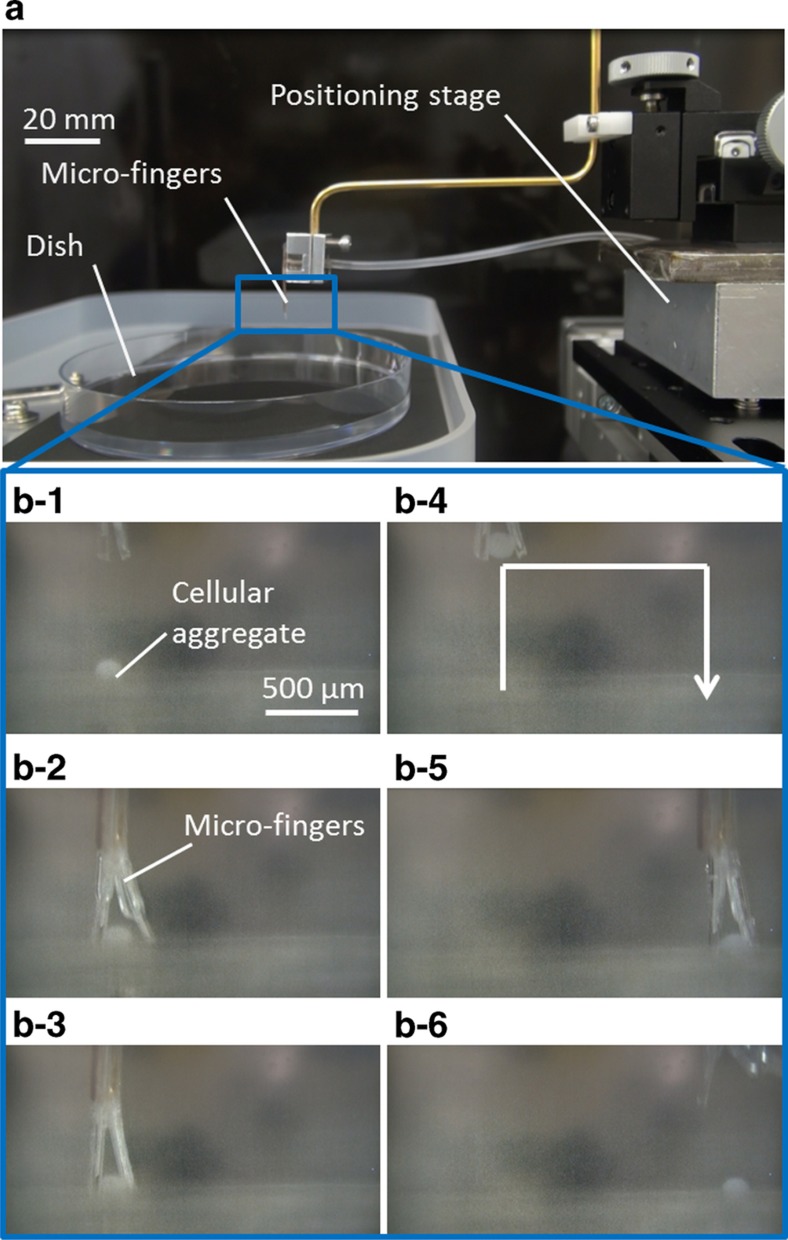
Manipulation of a cellular aggregate using a three-dimensional tracking stage system. (**a**) A whole view of the setup. (**b**) Series of operation using the tracking stage system: (**b-1**) initial state; (**b-2**) positioning; (**b-4**) pinching a cellular aggregate by microfingers; (**b-5**) moving to the destination; (**b-6**) releasing a cellular aggregate.

The cellular aggregate could also be transported to another desired well according to the design of the manipulation system. Figure 9 shows the experimental set-up and operational results of cellular aggregate manipulation. A cellular aggregate can be identified through the microscope, enabling the microfingers to be positioned towards the cellular aggregate. The following series of operations was possible using the positioning system: (1) initial state; (2) recognizing a cellular aggregate in a well or dish; (3) positioning the microfingers to a targeted cellular aggregate; (4) pinching a cellular aggregate by opening and closing the fingertips; (5) moving to the destination; and (6) releasing a cellular aggregate by opening the fingertips. The series of operations were performed successfully by the implemented microfingers and positioning system as shown in Figure 9.

## Conclusion

Soft microfingers were designed for manipulation of a cellular aggregate, these soft microfingers were implemented and evaluated in this paper. The contact surface of the microfingers was improved, and the manipulation system was implemented to accomplish a manipulation task. A bending type of PBA was applied to drive the microfingers by exploiting its small, soft and safe features. Two bending PBAs were bonded back-to-back to function as the microfingers. The fingertips closed normally and could be opened by pressurization. The dimensions of a single fingertip were 560 μm×900 μm.

Pinching of a cellular aggregate was evaluated to test the basic performance of the developed microfingers. The restoring force by pinching the microfingers against a *φ*200-μm cellular aggregate was estimated to be 30 μN. In the damage evaluation according to cellular cytotoxicity, no obvious damage was observed when applying up to 1 mN of restoring force. The result showed that the developed microfingers are suitable for cellular aggregate manipulation.

The combination of releasing and pinching of a cellular aggregate highlighted an initial problem of the cellular aggregate sticking to a fingertip. We solved this problem by improving the wettability condition of the surface of the microfingers. We examined various surface conditions and employed VUV treatment of the parylene-coated PDMS with a contact angle of 40°. Furthermore, a positioning system using an XYZ stage allowed for targeted positioning of microfingers for transport to a desired location. As a result, the series of operations including pinching and releasing could be successfully performed with the developed microfingers.

## Figures and Tables

**Table 1:**
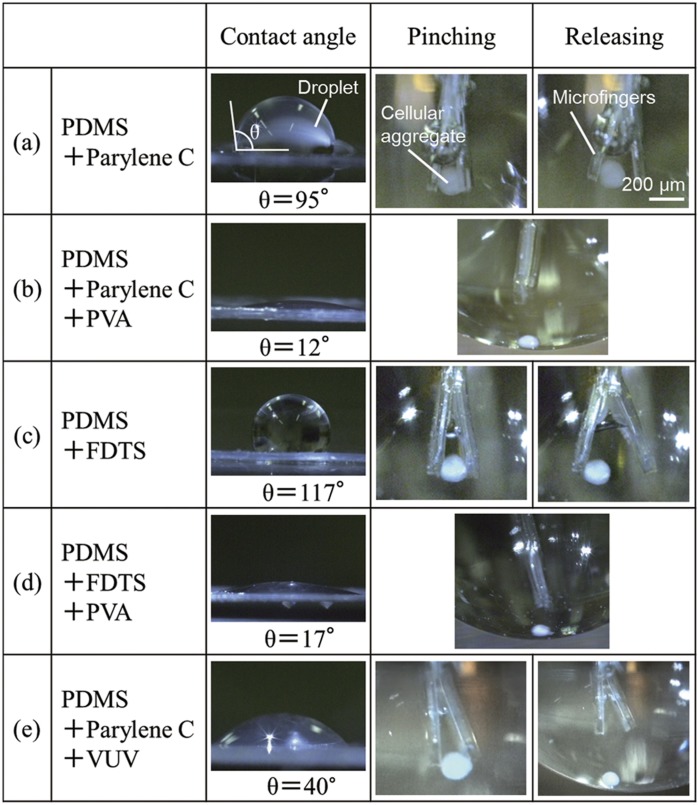
Surface wettability optimization for both holding and releasing cellular aggregate by microfingers

## References

[bib1] Hsu Y , Lucas K , Davis D et al. Novel Strain relief design for multilayer thin film stretchable interconnects. IEEE Transactions On Electron Devices 2013; 60: 2338–2345.

[bib2] Fuketa H , Yoshioka K , Shinozuka Yasuhiro et al. 1μm-thickness 64-channel surface electromyogram measurement sheet with 2v organic transistors for prosthetic hand control. 2013 IEEE International Solid-State Circuits Conference Digest of Technical Papers (ISSCC); 17-21 Feb 2013; San Francisco, CA, USA; 2013: 104–105.

[bib3] Khang D , Rogers J , Lee H . Mechanical buckling: mechanics, metrology, and stretchable electronics. Advanced Functional Materials 2009; 19: 1526–1536.

[bib4] Konishi S , Honsho K , Sugiyama S . Direct drawing for microfabrication without photolithography. The 12th IEEE International Conference on Micro Electro Mechanical Systems (MEMS'99); 21-21 Jan 1999; Orlando, FL, USA; 1999: 194–199.

[bib5] Konishi S , Honsho K , Yamada M et al. Direct drawing method for microfabrication based on selective metal plating technology. Sensors and Actuators A 2003; 103: 135–142.

[bib6] Lo CY , Kiitola-Keinanen J , Huttunen O-K et al. Micro roll-to-roll patterning process and its application on flexible display. Japanese Journal of Applied Physics 2009; 48: 06FC04.

[bib7] Solchaga LA , Dennis JE , Goldberg VM et al. Hyaluronic acid-based polymers as cell carriers for tissue-engineered repair of bone and cartilage. Journal of Orthopaedic Research 1999; 17: 205–213. 10221837 10.1002/jor.1100170209

[bib8] Hartgerink J. D , Beniash E. , Stupp SI . Self-assembly and mineralization of peptide-amphiphile nanofibers. Science 2001; 294: 1684–1688. 11721046 10.1126/science.1063187

[bib9] Hosseinkhani H , Azzam T , Kobayashi H et al. Combination of 3D tissue engineered scaffold and non-viral gene carrier enhance *in vitro* DNA expression of mesenchymal stem cells. Biomaterials 2006; 627: 4269–4278. 10.1016/j.biomaterials.2006.02.033 16620957

[bib10] Mohan N , Nair PD , Tabata Y . A 3D biodegradable protein based matrix for cartilage tissue engineering and stem cell differentiation to cartilage. Journal of Materials Science: Materials in Medicine 2009; 2009: 10.1007/s10856-008-3481-7. 10.1007/s10856-008-3481-7 18560767

[bib11] Vepari CP , Kaplan DL . Covalently immobilized enzyme gradients within three-dimensional porous scaffolds. Biotechnology and Bioengineering 2006; 93: 1130–1137. 16444737 10.1002/bit.20833

[bib12] Kurosawa H . Methods for inducing embryoid body formation: *In vitro* differentiation system of embryonic stem cells. Journal of Bioscience and Bioengineering 2007; 103: 389–398. 17609152 10.1263/jbb.103.389

[bib13] Yasukawa A , Ikeuchi M , Ikuta K . Combinatorial differentiation induction of embrionic bodies in PASCL (Pneumatically Actuated Spheroids Culture Lab-On-Chip). The 26th IEEE Iternational Conference on Micro Electro Mechanical Systems (MEMS'13); 20-24 Jan 2013; Taipei; 2013: 931–934.

[bib14] Teramachi Y , Shimomura S , Tonomura W et al. Cellular aggregate catcher using fluidic manipulation in high compatibility with wide spread -plate. The 26th IEEE Iternational Conference on Micro Electro Mechanical Systems (MEMS'13); 20-24 Jan 2013; Taipei; 2013: 2209–2212.

[bib15] Konishi S , Teramachi Y , Shimomura S et al. Cellular aggregate capture by fluidic manipulation device highly compatible with -plates. Biomedical Microdevices 2015; 17: 9953. 25846275 10.1007/s10544-015-9953-x

[bib16] Ok J , Lu Y-W , Kim C-J . Pneumatically driven microcage for microbe manipulation in a biological liquid environment. Journal of Microelectromecanical Systems 2006; 15: 1499–1505.

[bib17] Tanikawa T , Arai T . Development of a micro-manipulation system having a two-fingered micro-hand. IEEE Transactions on Robotics and Automation 1999; 15: 152–162.

[bib18] Jeong O , Konishi S . All PDMS pneumatic microfinger with bidirectional motion and its application. IEEE/ASME Journal of Microelectromecanical Systems 2006; 15: 896–902.

[bib19] Shimomura S , Teramachi Y , Muramatsu Y et al. Pinching and releasing of cellular aggregate by micro-fingers using pdms pneumatic balloon actuators. The 27th IEEE International Conference on Micro Electro Mechanical Systems 2014; 26–30 January 2014; San Fransisco, CA, USA; 2014: 925–926.

[bib20] Konishi S , Kawai F , Cusin P . Thin flexible end-effector using pneumatic balloon actuator. Sensors and Actuators A 2001; 89: 28–35.

[bib21] De Volder M , Reynaerts D . Pneumatic and hydraulic microactuators: a review. Journal of Micromechanics and Microengineering 2010; 20: 1–18.

[bib22] Lu Y , Kim CJ . Micro-finger articulation by pneumatic parylene balloons. TRANSDUCERS, Microsystems, The 12th International Conference on, Solid-State Sensors, Actuators and Microsystems (TRANSDUCERS 2003); 8-12 Jun 2003; Boston, MA, USA; 1: 276–279.

[bib23] Konishi S , Kobayashi T , Maeda H et al. Cuff actuator for adaptive holding condition around nerves. Sensors and Actuators B 2002; 83: 60–66.

[bib24] Fujimoto Y , Fujiwara N , Saito Y et al. Medical application with pneumatic balloon actuator to retracto system or endoscopic submucosal dissection. Proceedings of 27th Sensor Symposium 2010; 2010: 559–564.

[bib25] Shimizu K , Kawakami S , Hayashi K et al. *In vivo* site-specific transfection of naked plasmid DNA and siRNAs in mice by using a tissue suction device. PLoS ONE 2012; 7: e41319. 22844458 10.1371/journal.pone.0041319 PMC3402481

[bib26] Watanabe Y , Maeda M , Yaji N et al. Small, soft, and safe microactuator for retinal pigment epithelium transplantation. IEEE 20th International Conference on Micro Electro Mechanical Systems (MEMS 2007); 21-25 Jan 2007; Hyogo, Japan: 2007: 659–662.

[bib27] Tokida M , Obara T , Takahash M et al. Integration of cell sheet suking and tactile sensing functions to retinal pigment epithelium transplantation tool. IEEE 23th International Conference on Micro Electro Mechanical Systems (MEMS 2010); 24-28 Jan 2010; Wanchai, HongKong: 2010: 315–319.

[bib28] Okamoto T , Aoyama T , Nakayama T et al. Clonal heterogeneity in differentiation potential of immortalized human mesenchymal stem cells. Biochemical and Biophysical Research Communications 2002; 295: 354–361. 12150956 10.1016/s0006-291x(02)00661-7

[bib29] Gregoriades N , Clay J , Ma N et al. Cell damage of microcarrier cultures as a function of local energy dissipation created by a rapid extensional flow. Biotechnology and Bioengineering 2000; 69: 171–182. 10861396 10.1002/(sici)1097-0290(20000720)69:2<171::aid-bit6>3.0.co;2-c

